# Publisher Correction: Precision cosmology from future lensed gravitational wave and electromagnetic signals

**DOI:** 10.1038/s41467-017-02135-6

**Published:** 2017-12-12

**Authors:** Kai Liao, Xi-Long Fan, Xuheng Ding, Marek Biesiada, Zong-Hong Zhu

**Affiliations:** 1 School of Physics and Technology, Wuhan University, Wuhan, 430072 China; 2 School of Science, Wuhan University of Technology, Wuhan, 430070 China; 3 Department of Physics and Mechanical and Electrical Engineering, Hubei University of Education, Wuhan, 430205 China; 4 Department of Astronomy, Beijing Normal University, Beijing, 100875 China; 5 Department of Physics and Astronomy, University of California, Los Angeles, CA 90095-1547 USA; 6 Department of Astrophysics and Cosmology, Institute of Physics, University of Silesia, Uniwersytecka 4, 40-007 Katowice, Poland

*Nature Communications* **8**:1148 10.1038/s41467-017-01152-9; Article published online: 27 October 2017

The original PDF version of this Article inadvertently highlighted the author surnames and omitted the publication date. These have now been corrected in the PDF version of the Article. The HTML version was correct from the time of publication.

